# Examining Nurses’ Vengeful Behaviors: The Effects of Toxic Leadership and Psychological Well-Being

**DOI:** 10.3390/bs12110452

**Published:** 2022-11-14

**Authors:** Oktay Koç, Hayrettin Şahin, Gökten Öngel, Ayşe Günsel, Julie Aitken Schermer

**Affiliations:** 1 Political Science and Public Administration, Sinop University, Sinop 57000, Turkey; 2 Ministry of Health, Istanbul 34440, Turkey; 3 Management and Organization, Kocaeli University, Kocaeli 41040, Turkey; 4 Management and Organizational Studies, Faculty of Social Science, The University of Western Ontario, London, ON N6A 5C2, Canada

**Keywords:** leadership, personal satisfaction, mental health, administrative nursing research

## Abstract

Toxic leadership is becoming increasingly common in healthcare organizations and there is strong need for studies focusing on organizational factors that can trigger revenge. Additionally, how psychological well-being functions in shielding against toxicity has not been adequately studied. Hence, this study aims to examine the relationship between toxic leadership and vengeful behaviors of nurses, along with the contingency of psychological well-being on the relationship during the COVID-19 pandemic. In this exploratory cross-sectional study, we attempt to examine the antecedent effect of toxic leadership on vengeful behaviors based on self-reports from 311 nurses. Using partial least squares and moderation analyses, the results show that toxic leadership is an important antecedent of vengeful behaviors among nurses. However, the results provide no statistical evidence to support a moderating role of psychological well-being in the relationship between toxic leadership and vengeful behaviors. This study reveals that nurses exposed to toxic behaviors by their superiors are more likely to engage in vengeance and highlights the fact that nurses are suffering psychologically during the pandemic.

## 1. Introduction

Even though leaders are generally considered to be skilled, experienced and ethical in their behaviors, they can also be self-serving, arrogant and incompetent [1]. Although the focus on positive leadership still represents the mainstream in relevant studies, some recent studies argue that leadership also involves a dark side (i.e., destructive, abusive, despotic, narcissist and toxic leaders) that should be explored within the context of employee-level outcomes [2,3,4].

Toxic leadership emerges when a leader performs systematic and destructive behaviors that results in direct or indirect harm to others and the organization [5]. Toxic leaders create a demotivating, dehumanizing, fearful and unfair business environment [6]. Previous studies about toxic leadership reveal negative outcomes at the employee level, such as decreased representative fulfillment and responsibility, deterioration in health, demotivation, fear and perceptions of unfairness [5,7,8]. In addition, employees’ perceptions of toxic leadership will often lead to punitive evaluations of those leaders and vengeful behaviors [9,10,11].

Vengeful behaviors in the workplace represent counterproductive behaviors [12,13] which are punitive and damaging responsive behaviors to perceived wrongdoing [14]. Although there is a common consensus in the literature that vengeful behaviors have various negative and/or positive outcomes [15], scholarly works generally emphasize the negative outcomes that result from feeling unfairly treated [12]. In this sense, revenge may trigger counter-revenge, prolonged disputes [16] and is often seen in association with a variety of adverse psychological outcomes [17]. For example, Staub [18] argues that the desire for revenge disrupts an individual’s mental well-being, as well as their social relationships with others because of the aggressive and violent nature associated with revenge. Therefore, revenge has been conceptualized as an immoral, unethical and dysfunctional aspect of organizations [15].

In addition to the effects of toxic leadership upon an employee’s desire for vengeance, toxic leadership also has an influence on the employees’ job satisfaction and organizational commitment [19]. These are dimensions associated with psychological well-being, which are essential for individuals to succeed, mentally evolve and psychologically grow [20]. Psychological well-being is conceptualized as the mental capacity needed to solve problems, adjust to environmental changes and meet the expected targets [21]. Individuals with positive psychological well-being are less prone to negative feelings, attitudes or behaviors, even in unhealthy work environments [22]. In addition, some past studies [23,24,25] have demonstrated a negative relationship between vengeful behavior and psychological well-being. This relationship may suggest that psychological well-being may play a role in shielding or reducing the probability of vengeful behaviors after experiencing toxic leadership, because psychological well-being can result in the inducement of positive emotions [25]. Consequently, in this study, we hypothesize that excited and energized employees with positive psychological well-being [26] and a belief that they are contributing to something important will better adapt to toxic business atmospheres and are less likely to engage in vengeful behaviors.

### Present Study

In the present study, we aim to reveal the effects of toxic leadership upon employees’ vengeful behaviors. Moreover, we also try to reveal the possible moderating role of psychological well-being on the relationship between employee perceptions of toxic leadership and vengeful behaviors. To test our hypotheses, we collect data from a sample of nurses. Although the effects of negative leadership styles on employee-level adverse outcomes have been previously studied in both the organizational behavior and health management literature, there is still room for empirical studies that focus on the other organizational factors that can trigger revenge, such as toxic leadership [15]. Furthermore, the extant literature about toxic leadership was originally conducted with personnel from the military, political and business contexts [7]. However, as this type of leadership has become increasingly common in the healthcare industry [27], recent studies have begun to show more interest in examining this issue in healthcare organizations [28,29]. In this context, nurses constitute an important occupational group that is under pressure, and are occasionally exposed to dysfunctional, destructive and toxic management [30]. In addition, hospitals and healthcare institutions have been addressing the conditions associated with the COVID-19 pandemic. For this reason, nurses are more likely to encounter negative emotions, attitudes and behaviors, such as sadness, fear, neglect of work, harassment, bullying and even vengeful behavior during the pandemic period [31].

Prior research addressing toxic leadership in healthcare organizations has reported that when health workers, such as nurses, are confronted with toxic leadership, they are affected physically, emotionally and psychologically [32]. In addition to this, Labrague [33] has also reported that toxic leadership is one of the prominent reasons for nurse-reported adverse events and poor quality of care in their work units. The current study differs from past studies in that the focus is not simply on toxic leadership, but also on the possible role that psychological well-being may play on the effects of toxic leadership.

The following important two research questions substantially guide this study: (i) how do nurses’ perceptions of toxic leadership from their supervisors correlate with vengeful behaviors and (ii) how does the relationship between perceptions of toxic leadership and vengeful behaviors differ due to psychological well-being for nurses?

## 2. Methods

### 2.1. Study Design

The present study is cross-sectional and correlational by design.

### 2.2. Sample and Sampling Procedure

Nurses from six hospitals (three private and three public) located in Kocaeli were surveyed. According to The Ministry of Health of Turkey [34], 7312 nurses are actively employed in Kocaeli. Of those nurses, 600 were selected based on convenience sampling as our target. First, the chief physicians of the selected six hospitals were contacted and the purpose of the study was explained. The chief physicians provided a list of 100 nurses to be contacted (100 nurses from each of the six hospitals). Of the 600 nurses contacted, 457 accepted to join the study, and of those, 342 nurses completed the survey. After an examination of the survey responses, 31 surveys were discarded due to missing data. The final sample consisted of 311 nurses (88% women). Age information was collected using age intervals. For the sample, 141 (45.3%) of the sample was between 22 and 30 years old, 78 (25.1%) were between 31 and 40 years old, 85 (27.3%) were between 41 and 50 years old and the remaining 6 participants were over the age of 51 years. In terms of marital status, 63% were married. Moreover, 93% of the participants had a bachelor’s or higher degree, 85% of the participants were employed in general hospitals with more than 200 beds, and 46% had worked 1 to 5 years at the hospital.

### 2.3. Variables

In addition to completing questions regarding age range, biological sex, marital status, education and years of employment, participating nurses also completed three published scales.

#### 2.3.1. Toxic Leadership

Toxic leadership was assessed using the 30-item scale developed by Schmidt [3], which consists of the following five sub-dimensions: abusive supervision (example item: “Tells subordinates they are incompetent”), authoritarian leadership (example item: “Will ignore ideas that are contrary to his/her own”), narcissism (example item: “Thinks that he/she is more capable than others”), self-promotion (example item: “Will only offer assistance to people who can help him/her get ahead”) and unpredictability (example item: “Expresses anger at subordinates for unknown reasons”). In the present sample, the internal consistency (Coefficient alpha) value ranged from 0.089 for authoritarian leadership to 0.964 for unpredictability (see Table 1). The descriptive statistics (average item responses) are presented in Table 1.

#### 2.3.2. Vengeful Behavior

Vengeful behavior was assessed based on an adaptation of the 10-item vengeance scale (example item: “If I am wronged, I can’t live with myself unless I get revenge”) developed by Coelho et al. [35]. In the present sample, the internal consistency (Coefficient alpha) value was 0.940. The descriptive statistics (average item responses) are presented in Table 1.

#### 2.3.3. Psychological Well-Being

Psychological well-being was assessed using an eight-item scale (example item: “My social relationships are supportive and rewarding”) adapted from Diener et al. [36]. It had an internal consistency (Coefficient alpha) value of 0.929. To provide continuity, each item was responded to using a 1 to 5 response key. The descriptive statistics (average item responses) are presented in Table 1.

### 2.4. Scale Translations

For each of the measures described above, the standard translation-back translation method was employed.

### 2.5. Ethics

Ethics approval for this study was obtained from Kocaeli University’s Social and Human Sciences Ethics Review Committee (28 May 2021-E.62972). All participants provided informed consent and could withdraw from completing the survey at any time.

### 2.6. Statistical Methods

In addition to calculating descriptive statistics, internal consistency estimates and zero-order Pearson correlation coefficients, we employed the PLS-SEM approach for the path analyses because of numerous reasons. First, as Fornell and Larcker [37] address, PLS does not involve several limiting assumptions, such as distributional assumptions, caused by maximum likelihood techniques. PLS is a latent variable modeling method that integrates many dependent constructs and explicitly distinguishes measurement error. Moreover, PLS is not sensitive to sample size considerations and can appropriately work with small samples over thirty compared to covariance-based SEM [38].

## 3. Results

### 3.1. Measurement Validation

To evaluate the psychometric features of the measures, we created a null model without any structural relationships. We then estimated the composite scale reliability (CR), Cronbach alpha and average variance extracted (AVE) values to assess reliability. Based on these analyses, one item from the authoritarian leadership scale was removed as the item reduced the reliability of the scale. Dropping the item was found to not reduce the content validity of the construct. After dropping the item, the PLS-based CR was higher than the threshold value of 0.70, Cronbach’s alpha surpassed the threshold value of 0.70 and the AVE goes above the 0.50 threshold value for all the first order constructs (see Table 1).

In addition to the reliability estimates, the discriminant validity of the measures was examined, following the recommendations of Fornell and Larcker [37]. According to Fornell and Larcker [37], the AVE for each construct should exceed the squared correlation values among the constructs. Table 1 demonstrates the correlation amongst all first-order variables, suggesting discriminant validity. Such results imply that the items have more common variance with their relevant constructs than with the dimensions [39]. Finally, the convergent validity was estimated by examining the standardized loadings of the measures onto their relevant constructs. As demonstrated in the main diagonal in Table 1, each scale had standardized loadings greater than 0.60.

### 3.2. Toxic Leadership

Because we wanted to examine the overall effect of toxic leadership on vengeful behavior, we conducted a secondary factor analysis to generate a composite variable instead of examining each toxic leadership dimension individually. The standardized regression loadings of the sub-scales onto a toxic leadership factor were 0.87, 0.90, 0.85, 0.93 and 0.91 for abusive supervision, authoritarian leadership, narcissism, self-promotion and unpredictability, respectively. These results provide some empirical evidence in support of a composite toxic leadership scale score.

### 3.3. Path Analyses

**Figure 1:**
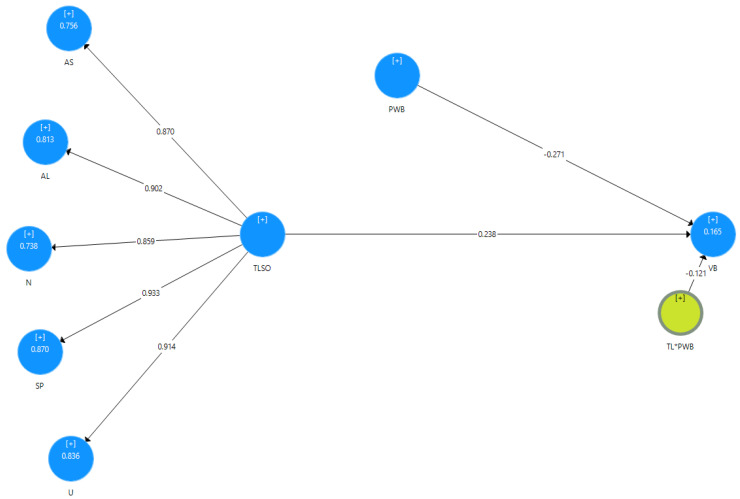
Path Analysis of Toxic Leadership and Psychological Well-Being predicting Vengeful Behaviors. Notes: AS = Abusive Supervision; AL = Authoritarian Leadership; N = Narcissism; SP = Self-Promotion; U = Unpredictability; TLSO = Second-order Toxic Leadership; PWB = Psychological Well-Being; VB = Vengeful Behaviors.

We utilized the partial least squares structural equation modeling (PLS-SEM) approach [40] with the bootstrapping re-sampling method, using the SmartPLS 3.0 statistical tool, to calculate the main and interaction effects between toxic leadership, vengeful behavior and psychological well-being. The path coefficients and their related *t*-values are presented in Table 2. They demonstrate a significant positive relationship between toxic leadership and vengeful behavior, with the full model represented in Figure 1. As Chin [40] recommends, we then employed a hierarchical approach for assessing the hypotheses and tested the contingency of psychological well-being on the relationship between toxic leadership and vengeful behavior via a two-step procedure [40]. The PLS technique enables the assessment of the standardized latent variable scores [41]. To avoid collinearity issues, we estimate the interaction terms by employing the product indicator approach [40]. The product indicator approach involves standardizing the items of constructs and calculating the interaction term through multiplying each item of one construct with all the items of the moderator. In this procedure, each item of toxic leadership and psychological well-being were standardized and then the items were multiplied. Surprisingly, our results did not provide any empirical evidence for the contingency of psychological well-being on the relationship between toxic leadership and vengeful behavior (see Table 2).

### 3.4. Structural Model

To confirm the validity of the PLS-SEM technique, numerous fit scores, including the coefficient of determination (*R*^2^) [40], the Q^2^ of predictive validity [42], and the standardized root mean squared residual (SRMR) [41], were calculated. Vengeful behavior (*R*^2^ = 0.17) had a medium effect size with satisfactory fit indicators (Q^2^ = 0.043, SRMR = 0.072).

## 4. Discussion

In this study, we attempted to contribute to the health management literature by offering a model for scholars and healthcare managers to understand potential interrelationships among toxic leadership, vengeful behavior and psychological well-being among nurses. This study is possibly the first to empirically investigate the potential shielding function of psychological well-being against the negative effects of toxic leadership in general, and particularly among the nurses during the pandemic.

The present results demonstrated that the perceptions of toxic leadership held by nurses is significantly and positively associated with self-report responses to questions asking about vengeful behaviors. Although leadership is often addressed as a positive force on followers and society [43], or as a positive and significant influence on performance [44], our findings underline that there might also be negative effects when individuals perceive their leaders as engaging in toxic leadership behaviors, resulting possibly in counterproductive behaviors by the employees [45,46].

As one of the significant representatives of dark leadership [47], toxic leaders play a critical role by affecting followers not only behaviorally but also psychologically [2] because of their leadership style [1]. Moreover, the effects on the health and well-being of followers from experiencing toxic leaders may be long term [46]. Interestingly, toxic leaders tend to undertake such a detrimental but purposive role because they are mostly self-serving, dysfunctional and/or weak [1]. In addition, toxic leaders create a demotivating, dehumanizing, fearful and unfair business environment [6]. Hence, employee reactions to toxic leadership styles, such as job dissatisfaction, resistance, absenteeism, revenge, intention to leave, a decrease in performance and counterproductive behaviors [43,46,48], have psychological, sociological and financial implications on the organization.

The present study tried to assess the possible moderating or shielding role psychological well-being may have on the relationship between perceptions of toxic leadership and vengeful behaviors [46]. Our findings did not provide any statistical support regarding the moderating role of psychological well-being. This research question requires further investigation as our results may have been dampened by the effects of the COVID-19 pandemic, which has had considerable negative affects upon employees’ psychological well-being, including increases in anxiety, depression nervousness and hopelessness [31,49].

### Limitations and Future Research

The first limitation to be noted deals with the responses to the instruments used in the study. When examining the mean values in Table 1, the responses to the scale items were on the lower side for each of the scales except for the measure of psychological well-being. These values may reflect a hesitancy on the part of the nurses to endorse negative measures, such as vengeful behaviors. Future research may include more positively worded items and examine multiple leadership style scales. The present study is also limited, such that the design was cross-sectional and the data were collected during a global pandemic. As suggested above, results of studies taken at less stressful periods of time may result in different conclusions.

In addition to the convenience sampling conducted in the present study, this study may be limited by the smaller sample size. We attempted to ensure homogeneity in the samples by choosing equal sample sizes from public and private hospitals. However, when compared to previous studies, our sample was smaller than the sample sizes utilized by Labrague [33], who had a sample of 926 nurses working at 20 hospitals, but larger than the samples from Özkan et al. [50], who investigated 244 nurses’ perceptions working at a university hospital, and Webster et al. [32], who surveyed 14 health care workers. To ensure greater generalizability of the results, future research may consider larger samples and include other health care workers in addition to nurses.

There may be a possible limitation to this study due to the common method bias, as the same participant who answered the dependent variable answered the independent variable. Thus, we tested our results against Kock`s [51] recommendation, which states that variance inflation factors (VIFs) higher than 3.3 indicate common method bias. The VIF analyses revealed that our VIF values ranged between 1.17 and 2.22, suggesting that the common method bias may not have a significant influence on the present results.

Vengeful behavior represents an important construct for future research. For instance, future studies may examine how specific human resources practices or organizational climate (e.g., empowerment, performance management, and organizational promotions and rewards) influence both the development and acceptance of toxic leadership and may possibly result into vengeful behavior on the part of employees. Future studies may also consider using other leadership approaches (i.e., transformational, masculine and feminine leaderships) to deepen our understanding regarding this rising, but relatively unexplored, concept of vengeful behavior.

## 5. Conclusions

This study reveals that nurses exposed to toxic leadership behaviors are more likely to engage in vengeful behavior. However, the findings do not provide any support regarding the moderating role of psychological well-being on the relationship between toxic leadership and vengeful behavior. These results may have been dampened due to the increased stress load experienced by nurses presently during the pandemic, and thus require replication in less stressful situations. Although this research only scratches the surface of the critical, yet underexplored subject, of vengeful behaviors, the study does highlight the need for leaders in the workplace to engage in civil behavior towards the employees under their supervision and span of control.

## Figures and Tables

**Table 1:** Discriminant validity, composite scale reliability (CR), average variance extracted (AVE), Cronbach alpha values and descriptive statistics.

Variables	SP	N	AL	PWB	VB	U	AS
SP.	0.884						
N	0.660	0.876					
AL	0.512	0.595	0.836				
PWB	−0.055	−0.083	−0.068	0.809			
VB	0.225	0.214	0.212	−0.139	0.807		
U	0.512	0.527	0.425	−0.074	0.241	0.908	
AS	0.658	0.621	0.597	−0.078	0.180	0.491	0.794
CR	0.947	0.943	0.92	0.938	0.949	0.97	0.922
AVE	0.782	0.768	0.699	0.654	0.651	0.824	0.630
alpha	0.931	0.924	0.893	0.929	0.940	0.964	0.904
Mean	1.671	1.785	1.583	3.825	1.743	1.717	1.455
*SD*	0.992	1.035	0.896	0.880	0.855	1.052	0.751

*Notes*: AS = Abusive Supervision; AL = Authoritarian Leadership; N = Narcissism; SP = Self-Promotion; U = Unpredictability; PWB = Psychological Well-Being; VB = Vengeful Behaviors.

**Table 2:** Path results.

Relationships			Path Coefficient (β)	Results
TL	→	VB	0.222 **	Supported
TL × PWB	→	VB	−0.022	Not Supported

*Notes*: TL = Toxic Leadership; PWB = Psychological Well-Being; VB = Vengeful Behaviors; ** *p* < 0.01.

## Data Availability

De-identified data is available by contacting the first author.
